# Changes in German Mental Health Care by Implementing a Global Treatment Budget—A Mixed-Method Process Evaluation Study

**DOI:** 10.3389/fpsyt.2020.00426

**Published:** 2020-05-25

**Authors:** Julian Schwarz, Laura Galbusera, Andreas Bechdolf, Thomas Birker, Arno Deister, Annette Duve, Philip Heiser, Kerit Hojes, Sonja Indefrey, Jakob Johne, Burkhard Rehr, Sandeep Rout, Harald Scherk, Anna Schulz-Du Bois, Bettina Wilms, Dyrk Zedlick, Manfred Zeipert, Martin Heinze, Sebastian von Peter

**Affiliations:** ^1^Department of Psychiatry and Psychotherapy, Brandenburg Medical School Theodor Fontane, Immanuel Klinik Rüdersdorf, Rüdersdorf, Germany; ^2^Department of Psychiatry and Psychotherapy, Vivantes Krankenhaus am Urban and Vivantes Klinikum im Friedrichshain, Charite University Medicine Berlin, Berlin, Germany; ^3^Department of Psychiatry and Psychotherapy, University of Cologne, Cologne, Germany; ^4^ORYGEN, National Center of Excellence of Youth Mental Health, University of Melbourne, Melbourne, VIC, Australia; ^5^Department of Psychiatry, Psychotherapy and Psychosomatic Medicine, Westklinikum Heide, Heide, Germany; ^6^Department of Psychiatry, Psychotherapy and Psychosomatic Medicine Psychosoziales Zentrum Itzehoe, Itzehoe, Germany; ^7^Department of Child and Adolescent Psychiatry, Vitos Klinikum Riedstadt, Riedstadt, Germany; ^8^Department of Child and Adolescent Psychiatry, Südharz Klinikum Nordhausen, Nordhausen, Germany; ^9^Department of Psychiatry and Psychotherapy, Südharz Klinikum Nordhausen, Nordhausen, Germany; ^10^Department of Psychiatry and Psychotherapy, Charité University Medicine Berlin, Berlin, Germany; ^11^Department of Psychiatry and Psychotherapy, Vivantes Krankenhaus Neukölln, Charité University Medicine Berlin, Berlin, Germany; ^12^Department of Psychiatry and Psychotherapy, Vitos Klinikum Riedstadt, Riedstadt, Germany; ^13^Department of Psychiatry and Psychotherapy, Imland Krankenhaus Rendsburg, Rendsburg, Germany; ^14^Department of Psychiatry and Psychotherapy, Basedow Klinikum Saalekreis, Querfurt, Germany; ^15^Department of Psychiatry and Psychotherapy, Rudolf Virchow Krankenhaus Glauchau, Glauchau, Germany

**Keywords:** global budget, capitation, block grant, integrated care, cross-sectoral mental health care, process evaluation, mixed method, complex intervention

## Abstract

**Background:**

Internationally, there is a broad spectrum of outreach and integrative care models, whereas in Germany acute psychiatric treatment is still mostly provided in inpatient settings. To overcome this, a new legal framework (§64b Social Code V) has been introduced, promoting “Flexible and Integrative Treatment” Models (FIT64b), based on a “Global Treatment Budget” (GTB) financing approach. 23 hospitals have implemented the framework according to local needs and concepts. Prior research has already identified specific components of FIT64b. Based on this, our paper aims to examine the implementation process and underpinning change mechanisms of GTB-based FIT64b models from a staff, service user and caregiver perspective.

**Method:**

31 focus groups and 15 semi-structured interviews were conducted with hospital staff (n = 138), service users (n = 63), and caregivers (n = 35) in 10 psychiatric hospitals implementing FIT64b. Using qualitative analysis, we identified 5 core themes describing the implementation process, which were theoretically modeled into a logical diagram. The core mechanisms of change were thus identified across themes. Additional structural and semi-quantitative performance data was collected from all study departments.

**Results:**

The qualitative analysis showed that the shift from a daily- and performance-based payment to a lump-sum GTB and the shift of resources from in- to outpatient settings were of crucial importance for the process of change. Saved budget shares could be reinvested to integrate in-, out-, and day-patient units and to set up outreach home care. Clinicians reported feeling relieved by the increase of treatment options. They also emphasized a stronger relationship with and a better understanding of service users and a simplification of bureaucracy. Finally, service users and caregivers experienced higher need-adaptedness of treatment, a feeling of deeper understanding and safety, and the possibility to maintain everyday life during treatment. Finally, two FIT64b implementation prototypes were classified according to the semi-quantitative performance data.

**Conclusion:**

Based on the results, we developed 3 core mechanisms of change of FIT64b models: (1) Need-adaptedness and flexibility; (2) Continuity of care; (3) Maintaining everyday life. Our findings outline and emphasize the potential a GTB approach may have for improving psychiatric hospital services.

## Introduction

Internationally, there is a broad spectrum of team-based outreach and integrative psychiatric care models dedicated to acute treatment (1–3). Yet, intensive psychiatric care in Germany is almost exclusively provided in inpatient hospital settings (4). To improve this situation, in 2013 a new legislation (§64b Social Code V) has been introduced to promote “Flexible and Integrative Treatment” Models (FIT64b). Importantly, FIT64b is a legal framework and no concrete model of care, leading to an implementation according to providers' specific context, needs and concepts. Nevertheless, all FIT64b models are based on a Global Treatment Budget (GTB), which is an annual lump-sum budget applied across all hospital settings (5). The GTB is negotiated between care providers and health insurances on the basis of historical expenditure and of the number of patients treated. Thus, this financing approach can be described as a middle ground between block contracts (in which providers are payed a fixed amount to deliver a specific, usually broadly-defined, service) and capitation (in which providers receive lump-sum payments based on the number of patients treated) (6, 7).

Evidence has shown that daily- and performance-based remuneration, which is the predominant financing approach for German psychiatric inpatient care, leads to treat service users (SU) as cost-intensively as possible, i.e., mainly in inpatient settings. This in turn also contributes to the fragmentation of meantal health care services, thus increasing the inpatient-outpatient gap (8). A GTB approach contrasts this tendency toward fragmentation by providing hospitals with the financial security and flexibility needed to develop more integrative, ambulatory and outreach psychiatric care (9). However, GTBs only partially address the problem of fragmentation as long as they are limited to the hospital sector.

To date, 23 German psychiatric hospitals have introduced FIT64b models based on a GTB. The first outcome evaluations of these models have already shown positive effects, such as a reduction in inpatient length of stay, as well as an increase in the number of patients treated in outpatient and outreach settings (10–14). Most importantly, clinical outcomes (e.g., HoNOS, CGI, GAF) improved and overall psychiatric care costs were kept stable or even decreased (10, 15–18).

Although such outcome studies have shown that the introduction of GTB may yield notable changes in conventional (i.e., inpatient) services, it is still not clear how such changes might be brought about. Previous research, evaluating the process of FIT64b models, has identified 11 specific program components, which were operationalized and validated (12, 14, 19). In addition to these first attempts at operationally defining FIT64b models, further research is required to understand how these overarching components are implemented and how their interaction may produce change. Therefore, the following research questions will be adressed:

1. How do FIT64b models work and what are their common mechanisms of impact?
2. How do these impact mechanisms vary depending on different FIT64b implementations?
3. What role does the financing approach play in the implementation of FIT64b models?

The exploration of this research questions is especially necessary in order to delineate the multivariate effects of these treatment models on clinical practices and on the experiences of SU and carers.

The UK Medical Research Council (MRC) guidance on evaluating complex interventions recommends modeling, both theoretically and empirically, how the intervention processes are associated with changes in outcome (20). Following this guidance, in this paper we aim to explore the nexus between structure, process and outcome in FIT64b models. Accordingly, we develop a logical diagram that unpacks the underpinning mechanisms of change, starting from the resources and inputs up to the impacts on the different stakeholders involved.

## Methods

### Design

The present study is part of the EvaMod64b Project, a multicentre study aimed at exploring the experiences and evaluations of FIT64b models from three stakeholders' perspectives (hospital staff, SU, carers). Using a Mixed-Methods approach, the EvaMod64b Project combined a standardized survey, routine hospital data, and a semi-quantitative and qualitative assessment (11–14, 19, 21). An overview of quantitative outcomes and of some preliminary qualitative findings has been reported in a first publication on this project (12). To deepen the understanding of the overarching impact mechanisms of FIT64b models and to explain how these in turn are influenced by the degree of (concrete) implementation, in the present study we have implemented a detailed analysis of (1) semi-quantitative data about the degree of implementation of the FIT64b specific components and (2) qualitative data on the experiences of FIT64b implementation and outcomes from a multi-stakeholder perspective. Based on these qualitative analyses, and incorporating a theoretical framework, a logical diagram displaying the change mechanisms of FIT64b models was developed. Semi-quantitative data was used to show how these mechanisms vary according to different FIT64b model implementations.

### Setting and Sampling

In 2015, leaders of the 15 psychiatric departments included in the EvaMod64b Project were asked to participate in this study. 13 of them (10 of adult psychiatry and 3 of child and adolescent psychiatry departments) agreed to do so. Due to the lack of comparability with adult psychiatry, child and adolescent psychiatry departments were excluded from this study and were evaluated separately (22). The 10 participating psychiatric hospitals are located in the German regions of Schleswig-Holstein (Heide, Itzehoe, Rendsburg), Saxony (Glauchau), Thuringia (Nordhausen), Lower Saxony (Lüneburg), Hesse (Riedstadt), Berlin (districts of Kreuzberg and Neukölln), and Brandenburg (Rüdersdorf).

In each of these psychiatric departments, SU, caregivers and hospital staff members were selected for participating in the qualitative study, whereas semi-quantitative data were collected only from hospital staff members. As the focus of this study is mainly on process rather than – or only to a limited extent – on outcome evaluation, a larger number of hospital staff participants in comparison to SU and caregivers were recruited.

Participants were selected purposely in order to ensure the highest possible heterogeneity (especially within the focus groups). This was ensured by a study employee on site, who recruited participants using a sampling plan containing a precise description of the selection criteria (see **Supplementary Material, Table S1**). For instance, staff members who had worked within a FIT64b model for a long as well as only for a short time were included. Accordingly, SU and caregivers who had made treatment experiences with (specific components of) FIT64b models for a long or a short period of time were selected. SU were included in the study only if, at the time of the survey, they were not in an acute phase of illness and if they had enough German language skills. Caregivers were selected with and without reference to the participating SU. Generally, participants were directly approached by the study employee and according to the selection criteria defined in the sampling plan. Sampling was continued until data saturation was met (see *Qualitative Data Collection* and *Qualitative Data Analysis*). The number of all participants approached in the qualitative study and the number of individuals who denied participation or dropped out have not been monitored. Further details concerning the inclusion criteria of the study participants can be found in the first publication on this project (11). The study was approved by the Ethics Committee of the Brandenburg Medical School (2016, No. S 7 a) and was conducted in accordance with the 1964 Declaration of Helsinki and its later amendments. All participants gave their informed written consent.

### Assessment of Semi-Quantitative and Structural Data

Semi-quantitative data about the degree of implementation of each FIT64b specific component was captured using a standardised questionnaire (12), which was developed within the scope of this project and was filled in by the managerial staff of each psychiatric department. The operationalization of each component was quantified and thus measured on a rating scale (see **Table 1**). Further methodological remarks on the grading process have been published elsewhere (11, 12). In addition, structural data (including basic data about the hospitals' catchment areas and funding approaches) were requested (see **Table 2**).

**Table 1:** FIT64b model components and their operationalization according to von Peter et al. (2019).

No.	Component	Operationalisation	Assessment
**I**	Shifting in- to outpatient setting *Shift of treatment from I^1^ toward D^2^ and/or O^3^*	Number of outpatient CoT^4^/total number CoT^4^ during EP^5^	
**II**	Flexible care management across settings *Unproblematic shift of SoT^6^ (prompt, little bureaucracy)*	Number of CoT^4^ using all three SoT^6^ during EP^5^/total number SoT^6^ Treatment D^2^, I^1^, and/or O^3^ in the same unit (ward, level etc.) Systematic steering of treatment beyond all SoT^6^ Application of SoT^6^ spanning roster and therapy plans	**Rating scale (0–2)**
		Number SoT^6^-spanning sessions (meetings etc.)	**Rating scale (1–3)**
**III**	Continuity of treatment team *Implementation of team- and individual-related continuity*	Percentage of staff working in more than one SoT^6^ (on a regular basis) Coordinated admission (coordinating staff member) Coordination of treatment by e.g. case manager, SoT^6^-spanning care Home treatment by I^1^- and D^2^- teams Outsourced PIA (outpatient department) team (not working in I^1^ or D^2^)	**Rating scale (0–2)**
**IV**	Multiprofessional cooperation *Intense multiprofessional cooperation*	Absolute number of mandatory sessions across all occupational groups	**Absolute number**
		Measure/action to optimize cooperation across all occupational groups	**Rating scale (0–1)**
		Training sessions multiprofessional cooperation	
		Number occupational groups working in home treatment (on a regular basis)	**Rating scale (0–2)**
**V**	Therapeutic group sessions across all settings *Therapeutic groups with members from all SoT^6^*	Number of group sessions open for all SoT^6^	**Rating scale (0–2)**
**VI**	Outreach home care *Multiprofessional treatment at home ≥ 1 week*	Number CoT^4^ with home-treatment/all I^1^-cases during EP^5^	
		Cars for home-visits	**Rating scale (0–2)**
**VII**	Involvement of carers *Caregivers as therapeutic tool*	“Network” or other forms of systemic dialog with caregivers and/or “carer-conference” and/or “caregiver groups”	**Rating scale (0–1)**
		Number of groups open for carers	**Rating scale (0–1)**
		Percentage of systemic training for staff/employees (e.g. open dialogue)	**Percentage**
**VIII**	Accessibility of services *Geographical accessibility and accessibility of teams*	Accessibility of services within one-hour drive 24-hours-accessibility of multiprofessional mental health team (not doctor on call or the like) Shuttle service for services users	**Rating scale (0–2)**
		Waiting list	**Reverse rating scale (1–0)**
**IX**	Sovereign steering of services *Freedom of therapeutic decisions*	Number of exeats ≥ 2 nights in a row during EP Number of exeats per service user/calendar week during EP D^2^ treatment as well during the night Rules according to contract in all matters concerning setting of treatment and length of treatment	**Rating scale (0–2)**
**X**	Cooperation across sectors *Cooperation with ambulant care systems*	Mutual scheduling and realizing of treatment with ambulant care systems (Social Code V) Mutual scheduling and realizing of treatment with social welfare system (Social Code XII)	**Rating scale (0–2)**
		“Community psychiatric network”	**Rating scale (0–1)**
**XI**	Expansion of professional expertise *Professionalisation of staff*	Multiprofessional training of staff concerning FIT64b models Measures to multiply knowledge about FIT64b models FIT64b models as part of appraisal interviews	**Rating scale (0–1)**
		Percentage of nurses/caregivers moderating group sessions	**Percentage**

^1^I, inpatient; ^2^D, day-patient; ^3^O, outpatient; ^4^CoT, case of treatment; ^5^EP, evaluation period; ^6^SoT, setting of treatment (outpatient, day-patient, inpatient).

**Table 2:** Structural data of the psychiatric departments, including socio-geographic data of the corresponding catchment areas and data about hospital funding (year: 2016).

	Hospital departments									
	A	B	C	D	E	F	G	H	I	J
**Catchment area**										
Settlement	rural	rural	rural	rural	urban	urban	metropolitan	metropolitan	urban/ rural	rural
Population density (inhabitants per km²)	124	93	124	119	342	525	13.819	7.301	665	95
Inhabitants (in tousand inhabitants)	131	135	270	85	130	330	281	328	425	235
**Hospital funding**										
Sponsorship^1^	public	public	public	public	non-profit	public	public	public	public	non-profit
Contract closing date; Start of FIT64b implementation	2014-1	2013-1	2013-1	2014-1	2013-1	2016-1	2016-2	2016-1	2014-7	2014-1
Budget share (%)^2^	100	100	100	100	100	100	10	8,5	33	25
Experiences with similar funding approaches^3,4^	+	+	+	+						
Reduction of hospital beds since introduction of a GTB^4,5^	+	+	+	+	+				+	

^1^Public or non-profit hospital organisation; ^2^Portion of the hospital budget which is negotiated according to §64b Social Code V with a selection or all involved health insurances; ^3^Existing experiences with a Global Treatment Budget according to §24 “Bundespflegesatzverordnung”, the §64b preceding legislation, valid from 2002-2009, offering hospitals a fixed annual budget for the duration of 5 years; ^4^Maximum expression of parameter = +; ^5^GTB, Global Treatment Budget.

### Qualitative Data Collection

**Figure 2** gives an overview of the qualitative research process. We used different formats to collect in-depth qualitative data. First, expert interviews were conducted especially with program managers and chief physicians in 10 study departments. This may be considered as appropriate for a comprehensive description of the implementation process of FIT64b models, since expert interviews enable to “address a potential interview partner in a specific role, as he or she has access to knowledge that is not exclusive but not accessible to everyone in the field of action” (23). Second, focus groups were conducted to examine changes in treatment practice, culture and ethos and perceived effects. The group process aims to overcome subjective rationalizations and psychological barriers and to uncover underlying beliefs and ideas (24). To consistently gain knowledge from a multi-stakeholder perspective, focus groups were predominantly set up with staff, SU, and caregivers.

To carry out the qualitative evaluation (**Figure 2**; *Step I*) a semi-structured interview guideline was developed (see **Supplementary Material, Table S2**), based on the thematic fields of the aforementioned 11 FIT64b specific components.

Data was collected sequentially between April and October 2016 by SP together with one of the co-authors (changing for each specific site). In this period each of the 10 study departments was visited for 2–3 days. A total of 31 focus groups (2–5 in each department) and 15 expert interviews (1–3 in each department) was carried out. The average duration of the interviews and focus groups was approximately 84 min. Data collection was digitally recorded, transcribed verbatim, and anonymized. The analysis process started while data was still being collected. Regardless of the occurrence of theoretical saturation, data collection was performed in all departments.

### Theoretical Framework

**Figure 1:**
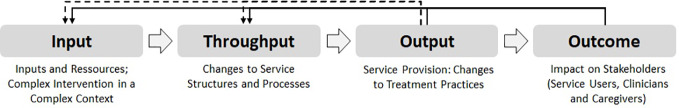
Throughput-Model, adapted from Pfaff and Schrappe (23).

Among various theoretical models that have been applied within this research field (see, e.g., 25), we selected the German Throughput-Model by Pfaff and Schrappe (26, 27) as a guiding framework due to its compatibility with the aims and nature of this study. Pfaff and Schrappe's (26) model draws on Donabedian's Structure-Process-Outcome theory (28), and it provides a solid and helpful framework for describing complex interventions, for clarifying their causal assumptions and for developing a program theory. **Figure 1** depicts a basic diagrammatic representation of the relationships between an intervention's *Input*, *Throughput*, *Output*, and intended *Outcomes* according to Pfaff and Schrappe (26).

*Input* does not only include the concrete resources needed to realize an intervention, but it also involves changes in regulations and conditions within the wider context of the health care system. These *contextual factors* may be interventions at system's level that, for instance, are followed by changes in hospital remuneration or legislation. *Throughput* describes changes in the structures and processes of an intervention (29) and *Output* corresponds to the level of service provision; it describes for example professional behaviors, organizational change, and possible changes within the health care system. *Outcome* describes the results of an intervention both at the stakeholders' and system's levels (27). Going beyond Donabedian's unilateral concept, the Throughput-Model puts forward a more systemically informed understanding of interventions: indeed, here, *Outcome* and *Output* are conceived as having a feedback function on *Input* and *Throughput*.

### Qualitative Data Analysis

**Figure 2:**
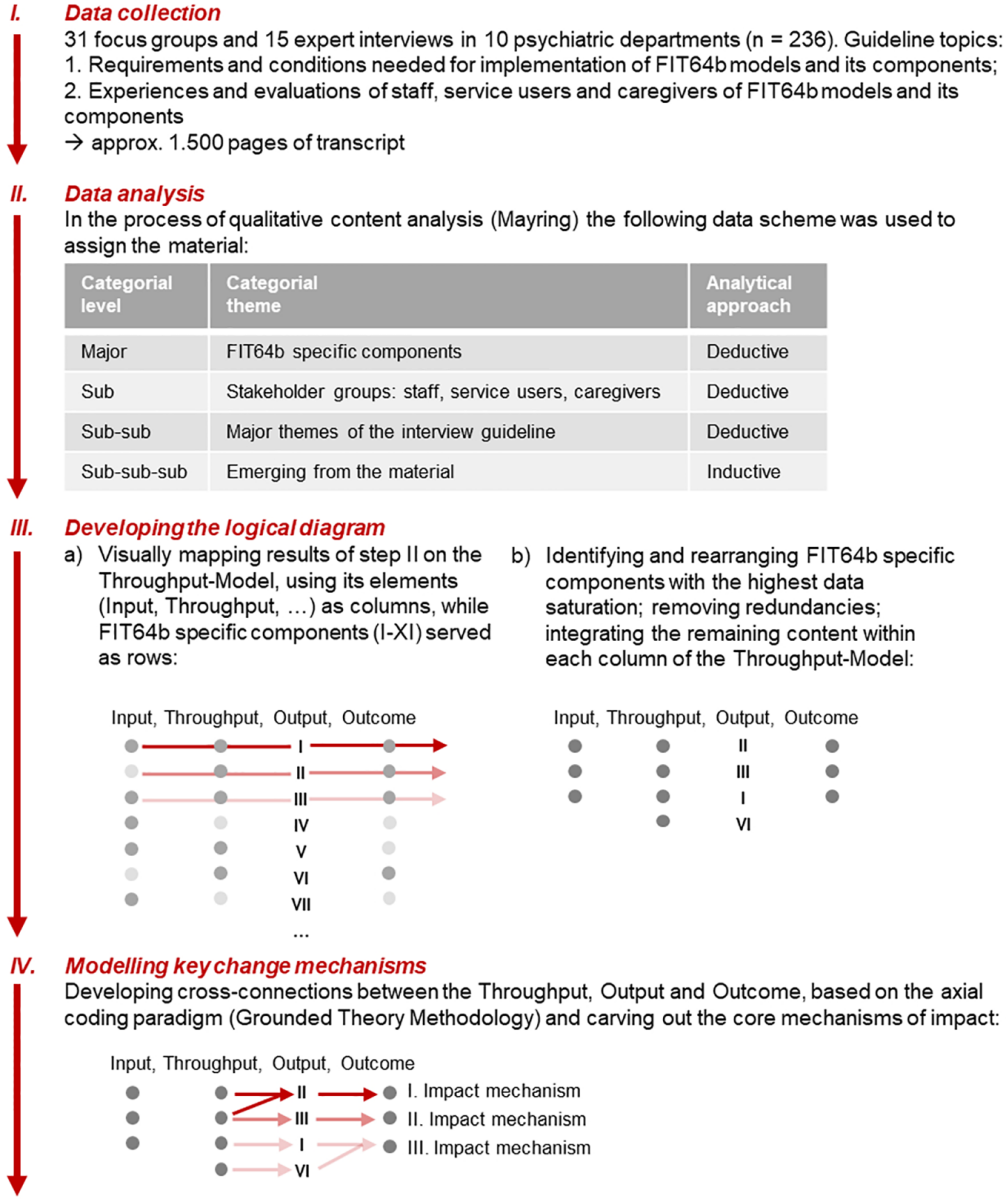
Process of qualitative data collection, analysis and modeling.

The first analytical step (**Figure 2**; *Step II*) was guided by the rule-based approach of content analysis (Mayring) (30, 31). We chose this methodology, because it provides a solid framework to transform great amounts of qualitative data into a more compact and reduced form yet conserving the original richness of information. Due to the extent of the material (approximately 1,500 pages of transcript) and in order to increase reliability, data were thematically split within the research team: Tandems of two researchers examined the material of each stakeholder group (SU, carers, staff) adopting a mixed deductive-inductive approach (**Figure 2**; *Step II*). In the process of content analysis, the specific components of FIT64b served as deductive major categories and analytic grid to which the qualitative material was assigned. Subsequently, the requirements and conditions needed for implementation of FIT64b models, the perceived changes in treatment practices, culture, and ethos and the effects of FIT64b models were analyzed and developed into further categories (**Figure 2**; *Step II*). Throughout this process the paired researchers (tandems) continuously met to discuss and to reach agreements on the intermediate and final categories. The full research group also worked together in several analytical workshops (“Forschungswerkstatt”) to triangulate and validate results.

### Logical Diagram and Key Change Mechanisms

In order to develop a logical diagram describing common aspects of FIT64b model implementation, JS and SP mapped the results of the prior analysis onto Schrappe and Pfaff's theoretical framework (**Figure 2**; *Step IIIa*) (26). In this process, the elements of the Throughput-Model (Input, Throughput, Output, Outcome) were used as columns, whereas the 11 FIT64b specific components served as rows. The results from the previous analytical step were thus gradually placed and arranged on this grid. Due to the great extend of the data the logical diagram had to be reduced, integrated, redundancies were removed and only those FIT64b specific components that showed the highest data density within the logical diagram were left (**Figure 2**; *Step IIIb*): These components were: (I) shifting from in- to outpatient settings, (II) flexible care management across settings, (III) continuity of treatment team, and (VI) outreach care. This is in accordance with the published results of a previous pilot study and with the quantitative findings of the EvaMod64b project, thus indicating that these four components can be considered as key aspects of FIT64b models (12, 32).

In a final analytical step (**Figure 2**; *Step IV*) we started searching for cross-connections between the elements of the Throughput-Model, applying the axial-coding paradigm of Grounded Theory Methodology (33). In an iterative process, three common impact mechanisms of FIT64b models could be carved out, leading from the Throughput to the Output and Outcomes. For the sake of clarity, change mechanisms were only made visible at the Throughput-level, because all elements of the Input and of the Throughput diverged from one another. During this analytical process, preliminary versions of the logical diagram were validated by the entire research team. Their suggestions for revisions were considered and the model was changed accordingly.

## Results

### Semi-Quantitative Findings

The semi-quantitative findings about the degree of implementation of FIT64b specific components are presented in **Table 3**. As the qualitative material presented below is limited to the key components (I, II, III, VI), the semi-quantitative findings are also limited to these.

**Table 3:** Implementation of FIT64b key components in the psychiatric departments (year: 2016).

FIT64b key components	Hospital departments									
	A	B	C	D	E	F	G	H	I	J
***I: Shifting service users from in- to outpatient settings***										
Number of outpatient CoT^1^/total number SoT^2^ during EP^3^ (%)	55,77	47,22	32,29	61,37	53,10	69,93	71,88	x^4^	60,62	43,37
***II: Flexible care management across settings***										
Treatment D^5^, I^6^, and/or O^7^ in the same unit (ward, level etc.)^8^	++++	++++	++	+++	+++	++		++	++	++
Systematic steering of treatment beyond all SoT^2,8^	+++	++++	++	+	+	+	+	+	+	++
Number SoT^2^-spanning sessions (meetings etc.)^8^	++++	++++	++		+++	++		+	++	+
Application of SoT^2^-spanning roster and therapy plans^8^	++++	++++	++	++	++++	++	++	+	+++	++
***III: Continuity of treatment team***										
Percentage of staff working in more than one SoT^2^ (on a regular basis)	>66%	>66%	>66%	>33%	>66%			>66%	>33%	>33%
Coordinated admission (coordinating staff member)^9^		+	+	+			+		+	
Coordination of treatment by e.g. case manager^9^		+	+	+	+			+	+	
Outreach home care by I^6^- and D^5^-teams^10^	+	++	++		+				+	
Outsourced outpatient department team (not working in I^6^ or D^5^)^9^	+									
***VI: Outreach home care***										
Implementation of outreach home care^9^	+	+	+	+	+		+	+	+	
Corresponding outreach care model^11^	ACT	ACT/CRT	ACT	ACT	ACT		CRT	CRT	ACT/CRT	
Number of cars	1	4	2	1	2		2	1	3	

^1^CoT, case of treatment; ^2^SoT, setting of treatment (outpatient, day-patient, inpatient); ^3^EP, evaluation period; ^4^x, data not provided; ^5^D, day-patient; ^6^I, inpatient; ^7^O, outpatient; ^8^ Maximum expression of parameter = ++++; ^9^Maximum expression of parameter = +; ^10^Maximum expression of parameter = ++. ^11^Assertive Community Treatment (ACT) or Crisis Resolution Teams (CRT).

### Qualitative Findings

A total number of 63 SU, 35 caregivers, and 138 hospital staff members were interviewed. **Table 4** shows the sociodemographic data of the study's participants.

**Table 4:** Participants' sociodemographic data.

Stakeholder group	n (%)	Female gender n (%)	Additional parameters
Service user	63 (26.7)	36 (57.1)	Ø 6,8 years duration of illness; n=24 (38.1%) currently in psychotherapeutic treatment; all psychiatric diagnoses were included with a focus on various forms of schizophrenia spectrum disorder
Caregiver	35 (14.8)	21 (60.0)	Ø 6,7 years duration of treatment of the respective relative; different kinds of caregivers were included, with a majority of mothers.
Staff	138 (58.5)	82 (59.4)	n=90 (65.1%) have worked in the same psychiatric department before the introduction of the GTB; n=48 (34.9%) had been working in other psychiatric hospitals before the introduction of the GTB.

GTB, Global Treatment Budget.

**Figure 3:**
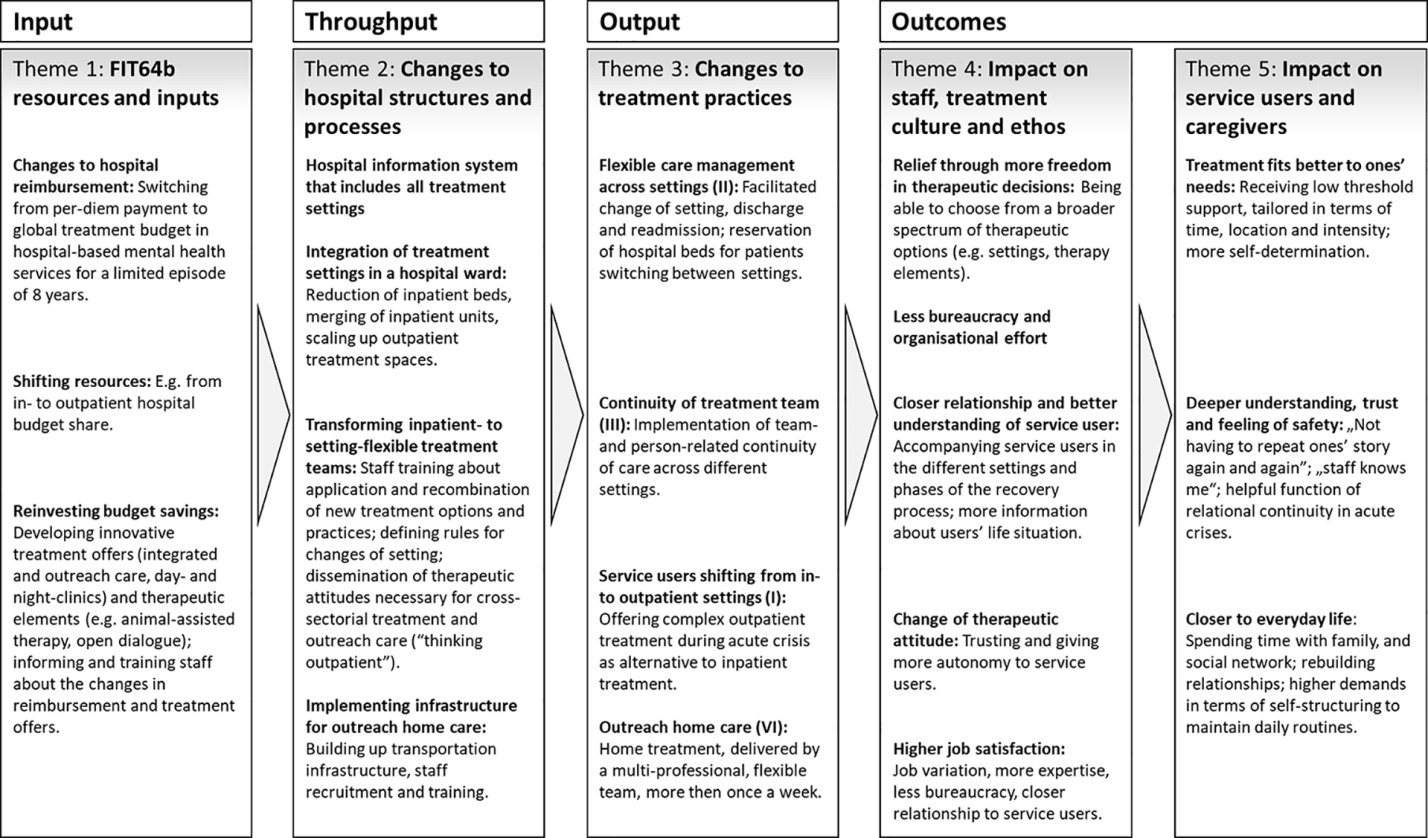
Logical diagram of FIT64b models, at once categorial system of the present qualitative process evaluation. I, II, III, VI: the Roman numerals refer to the key components of FIT64b. All FIT64b specific components can be found in **Table 1**.

As a result of content analysis, we carved out 5 core themes related to the implementation of the FIT64b, which were mapped onto the Throughput-Model (see **Figure 3**). The first three themes were labeled: *(I) FIT64b resources and inputs*; *(II) Changes to hospital structures and processes*; *(III) Changes to treatment practices*. Since these themes have a mainly descriptive character, we did not deem it necessary to report quotes from the transcripts. The last two themes were labelled: *(IV) Impact on staff, treatment culture and ethos*; *(V) Impact on service users and caregivers*. These themes entailed an evaluative aspect and are thus supported, in the presentation of results, by textual quotes from the transcripts.

#### Theme 1—FIT64b Resources and Inputs

In all the participating psychiatric departments, staff reported that the initiative to enter a contract according to §64b (German Social Code) came from both psychiatric hospitals and health insurances. Health insurance companies were often motivated by the prospect of controlling costs, whereas clinicians saw the possibility of maintaining predictable and constant compensation, which would facilitate the further development of psychiatric services.

##### Changes to Hospital Reimbursement

All the participating hospitals negotiated a fixed, lump-sum budget per annum (GTB) dedicated to financing the acute psychiatric care provided by either all or by only specific health insurances for a maximum term of 15 years. Compared with the usual performance-based remuneration in psychiatric hospitals, which leads to an increase in bed occupancy to maximize remuneration, a GTB would strengthen the tendency to act more proactively or preventively in order to avoid high resource usage.

##### Shifting Resources

Large parts of the hospital budget, which had previously been used to finance inpatient treatment structures, were moved to the outpatient sector. Since the average daily costs of inpatient care are significantly higher than those of outpatient treatment, hospitals could increase the intensity of outpatient work without having surplus costs.

##### Reinvesting Budget Savings

Hospitals reinvested the saved budget shares in order to act in accordance with the legislation. Study participants expressed quite different opinions about the use of saved budget shares. In general, these were invested for the further development of hospital structures (e.g., for outreach and integrated care, day and night clinics), for developing new therapeutic offers (e.g., animal-assisted or art therapy), or for training hospital staff (e.g., training in the systemic approach of Open Dialogue) (34).

#### Theme 2—Changes to Hospital Structures and Processes

In what follows, we present the key changes to hospital structures and processes, which were based on the intervention inputs and which were realized across all the participating psychiatric departments.

##### Hospital Information System That Includes All Settings

The hospital information system (HIS), which had so far processed in- and outpatient treatment separately, was merged to assure that (1) the clinical and performance documentation of each treatment setting could be accessed by the other settings and (2) patients could easily and flexibly shift from one setting to the other.

##### Integration of Treatment Settings in a Hospital Ward

In order to structurally integrate out-, day-, and inpatient treatment within one unit, areas that were previously used for patients' rooms were transformed into outpatient recreation and therapy rooms. As FIT64b models progressed, the proportion of patients treated in integrated out- and day-settings increased so that areas dedicated to inpatient treatment could be further reduced and inpatient wards could be closed or merged. Such restructuring was primarily implemented by the psychiatric departments A–D, that negotiated their entire hospital budget as a FIT64b model and that had prior experiences with a GTB. In three departments (A, F, J) new buildings were constructed in order to meet the FIT64b requirements and to allow the separation of sleeping and recreation areas, which were previously joined.

##### Transforming Inpatient- to Setting-Flexible Treatment Teams

Before the introduction of the FIT64b model, each staff member's clinical work was restricted to *one* setting. This regulation was dropped with the introduction of FIT64b: “setting-specific” teams were restructured into “flexible teams,” acting across all treatment settings (departments A–D). Therefore, processes and clinical routines, such as, planning therapies, shifted from being performed within specific settings to being extended across them. In FIT64b models, employees working on an inpatient ward kept flexible time slots available for outpatients. The departments (G–J) that introduced a GTB for just a part of their SU (less than ⅓ budget shares) tended to retain traditional team structures and established additional dedicated teams to exclusively attend to these SU.

To support these transformations, employees received several trainings aimed at promoting favorable attitudes toward “flexible and outpatient thinking.” Given that most staff members were trained within traditional hospital wards, a new approach toward mental health crises had to be introduced and taught. An important part of this process was the definition of clear and stable criteria for the change of setting, in order to facilitate the organisational change from a highly structured single-setting treatment, to a more unbound therapeutic work across settings.

##### Implementing Infrastructure for Outreach Home Care

Several structural requirements had to be fulfilled in order to allow the introduction of outreach forms of care. A business plan was needed in order to proof the feasibility of outreach home care within the hospital budget (share). New equipment, such as cars and mobile phones, was acquired and new solutions for mobile documentation and synchronisation with the HIS were developed. In rural catchment areas (clinics B–D, I) cars were purchased, whereas urban teams also used bicycles or public transport (clinics G, H). Employees received specific training for outreach work. Outreach work was realized either by (flexible) inpatient teams (clinics A–E) or by dedicated teams (clinics G–I). Due to extensive requirements, the component “outreach home care” was introduced with a delay of one to two years in the FIT64b model runtime.

#### Theme 3—Changes to Treatment Practices

The interaction of input and throughput factors lead to changes in service provision and treatment practices. These changes are best described by the developed processual and structural components of FIT64b models (**Table 1**). As mentioned above, we here only present 4 key components (I, II, III, VI) that reached data saturation during the process of thematic analysis. The reference to each specific component is indicated in the subheadings.

##### Flexible Care Management Across Settings (II)

Based on the GTB, new forms of support were introduced that involved flexible “degrees” of treatment intensity and the possibility for SU to flexibly shift between settings. As a result, SU who mistrusted inpatient psychiatric treatment could be slowly introduced to it by gradually increasing the treatment intensity. Furthermore, the increased overlaps between treatment settings allowed more flexible transitions to a SU's own home or workplace after inpatient care by gradually reducing the days or time of treatment (instead of ending it abruptly). The psychiatric departments A and B reserve inpatient beds during the phase of outpatient care to allow for a rapid admission in case of symptoms worsening. One clinic (J) introduced an acute day-patient setting for an uninterrupted day-treatment (also on weekends), whereas clinic B launched a night-patient setting for SU who need assistance only at night.

##### Continuity of Treatment Team (III)

The psychiatric departments A–E, which already had experience with previous FIT64b care and reimbursement models, achieved the highest degree of team- and person-related continuity of care (see **Table 3**). This is organized in various ways: either employees attend to their patients across various settings (departments A–D) – sometimes even in their homes in the case of outreach treatment teams (departments A–C) – or case managers were hired for coordinating the treatment process and thus ensuring continuity (departments B–E). In addition, some of the participating psychiatric departments (E, F) introduced adolescent psychiatry counseling teams, therefore also aiming at enabling smooth transitions into adult mental health services.

##### Service Users Shifting From In- to Outpatient Settings (I)

By integrating in- and outpatient settings, a significant portion of previously inpatient SU is now being treated within various outpatient settings, even during episodes of acute crisis. The psychiatric departments use outpatient facilities to prevent inpatient stays, to offer aftercare and to provide low-threshold access to inpatient forms of treatment.

##### Outreach Home Care (VI)

Following the introduction of a GTB-based accounting system, eight out of ten participating psychiatric departments currently offer multi-professional outreach treatment. Home visits are delivered on weekdays between 8–18 o'clock. The frequency of home visits (from daily to once every four weeks) and the duration of treatment (from < 2 to > 12 weeks) vary considerably between the departments. Departments in urban catchment areas are more likely to deliver shorter and high intensity treatment, whereas departments within rural areas provide longer treatment periods with less frequent visits.

#### Theme 4—Impact on Staff, Treatment Culture, and Ethos

Changes to hospital structures, processes, and treatment practices had complex impacts on the treatment culture and on the underlying therapeutic stance of employees.

##### Relief Through More Freedom in Therapeutic Decisions

The possibility and freedom to combine a broader range of therapeutic options and to take decisions about the course of treatment was described by employees as a relief and as a gain in therapeutic autonomy. By being less bound to the contingencies and restrictions of a specific setting, clinicians could tailor treatment more to the SU's needs:

*“So, if someone gets admitted and you notice after 2, 3 days, that he may benefit more from the day hospital setting, then we switch. And when it turns out that it was a bit risky, we can easily go back to inpatient conditions without having to clear up many formalities. This is very relieving for us but also for the patients” (Physician, Department B)*.

Staff members reported that they are currently free to decide how much time they intend to dedicate respectively to inpatient and outreach work. Hospital staff is also no more accountable for justifying the length of stay or the type of treatment to the health insurances. This also considerably contributed to the feeling of relief on their part.

Nevertheless, the employees participating in this study also described adverse effects of the increasing flexibility in the treatment process. In contrast to the therapeutic activities in regular care being usually limited to one setting,

*“people [both staff and SU] now have to be familiar with the peculiarities of in-, day-, outpatient and eventually also outreach work at the same time” (Nurse, Department B)*.

The increasing complexity of therapeutic options has been described by one employee as “*stressful freedom*” *(Nurse, Department B)*, as it yields more difficult decision-making processes.

##### Less Bureaucracy and Organizational Effort

The reduction of bureaucracy in FIT64b models played an important role within our data. In particular, the streamlining of documentation routines accompanying changes of setting was emphasized. The spatial integration of the treatment settings facilitated not only the exchange of information among staff but also the performance of everyday routines (by e.g. shortening distances):

*“Organizationally, my day was even easier: I do not have to change rooms to go to the day treatment unit or ambulance. I just stay in the same place” (Nurse, Department A)*.

In contrast, the organisation of group therapy sessions across all settings was described as a challenge: Since SU from different settings participated in the same group sessions, these groups were sometimes experienced by staff members as being too heterogeneous. Consequently, staff members reported difficulties in keeping track of the different setting (e.g., who is inpatient or outpatient) and in integrating participants with different needs.

##### Closer Relationship and Better Understanding of Service Users

The continuity of treatment across different settings promoted more stable relationships with SU and more comprehensive understanding of them and of their situations. This results from the fact, that SU are currently attended by the same therapist or therapeutic team during longer treatment episodes (both in- and outpatient), and not only during moments of acute crisis:

*“There's quite another level there, a level of trust and you do not have to start from scratch again. When the patient changes to day or outpatient treatment, you may discover a lot more about his or her resources, of which you then also may make use of. And this makes the treatment process more intense” (Social worker, Department A)*.

This allows staff members to attend to their patients through the different stages of the recovery process, thus supporting and facilitating the co-construction of shared solutions for complex problems. Yet, the trade-off of continuity is an increased difficulty in ending the therapeutic relation for both staff and SU:

*“Some patients don't find it easy to say goodbye to their reference therapist at the end of treatment. For longer courses, we therefore try to plan discharge at an early stage” (Psychotherapist, Department C)*.

Expanding therapeutic continuity beyond inpatient treatment to outreach and outpatient settings also allowed employees to develop a deeper understanding of the SU's life situation. In this regard, caregivers played an important role as sources of information, often empowering and mediating the relationship between SU and the treatment team.

##### Change of Therapeutic Attitude

Driven by the broader and more flexible spectrum of therapeutic options available and by the stronger therapeutic alliance, employees described an increased tendency to leave SU more autonomy:

*“Over the past few years we began to discharge patients earlier. Thereby, we have increasingly developed trust even to rather unstable patients – to clients that we would have kept in the ward in the past” (Nurse, Department B)*.

Employees' stronger orientation at outreach and outpatient care also contributed to their increased reliance on SU's competencies and resources:

*“I've been thinking a lot about how to improve my patients' resources. The more resources you develop during the patient's inpatient stay, the greater is the likelihood that an outpatient setting will work for him or her” (Psychologist, Department J)*.

Finally, the attitude toward caregivers also changed: caregivers are currently involved as active partners since the early stages of the treatment process instead of being considered as a mere source of information:

*“Relatives are less likely to be a resource on the ward and this is reversed in the home environment” (Nurse, Department G)*.

##### Higher Job Satisfaction

Overall, employees were satisfied with the new work models. They mentioned an increased motivation that resulted from their active involvement within this innovative model of treatment. For instance, additional therapeutic tasks were assigned to professional groups that traditionally did not work therapeutically. Such changes were perceived to increase therapeutic expertise, especially among the professional group of nurses. Yet, the increasing complexity of care pathways also led some employees to feel overwhelmed. Other employees critically noted that the additional therapeutic tasks were not appropriately remunerated. In general, however, employees' expressions of satisfaction outweighed their criticisms:

*“To be able to accompany a person through various phases: I experience this as enriching for me personally, but also for my profession. To accompany development, to see people grow. To see them going through crises, and still seeing that life goes on” (Physician, Department B)*.

#### Theme 5—Impact on Service Users and Caregivers

Changes to the treatment practices, the treatment culture and the underlying therapeutic stance of employees has led to several effects on SU and caregivers, which are described below.

##### Treatment Fits Better to One's Needs

From the SU's point of view, the increased flexibility of treatment in FIT64b models also led to its better adaptation to their needs. For instance, SU experienced treatment to be less oriented by institutional routines and instead to be more shaped around the concrete needs of their work or family life. Being granted the possibility to participate in decisions about when, where and at which intensity one gets support, seemed to relieve SU. A key factor within this sub-theme was the importance of and the preference for low-threshold support in acute situations:

*“With this [FIT64b] model, it was very flexible. I could say ‘Tomorrow I'll come from then till then' or ‘I'd rather sleep here [in the hospital]'. I could always just look and ask myself ‘How is it? What do I need now?' And then I got exactly the right help” (SU, Department B)*.

*“There was a note on the bed' Ms. X'. And if for once I could not handle a day at home, I could just move in here again” (SU, Department A)*.

##### Deeper Understanding, Trust, and Feeling of Safety

Mirroring and confirming employees' experiences (see Theme 4.2), SU and caregivers reported having felt better understood by the staff due to the continuity of treatment. This was experienced as a relief (“*not having to repeat ones' story again and again*”; *SU, Department C*). The awareness of having a constant reference therapist (or team), who is well informed on the situation and on what might help during crises, yielded feelings of trust and safety in SU and caregivers.

Comparable experiences were also reported by SU, who received outreach care: “*One feels safer at home than here in the hospital” (SU, Department I)*. Therapy time felt more intense within outreach forms of care; it was perceived by SU as being characterized by greater and more “undivided” attention by professionals, if compared to the inpatient setting. Furthermore, outreach care led to a change of traditional roles (patient as host; therapist as guest) that enabled more balanced power relations:

*“We spent some time hanging around in the kitchen together. This is my favourite place to sit and talk, whether for tea or for dinner. And that gave me the feeling, that yes, one speaks to me at eye level” (SU, Department H)*.

##### Closer to Everyday Life

A key outcome of FIT64b, defined by SU and caregivers, was the fact that this treatment model allowed them to carry on with their everyday-life activities also during moments of acute crisis:

*“That I can keep my usual environment and continue my everyday life while being treated at home, that is the most important thing” (SU, Department G)*.

Especially, integrated outpatient and outreach forms of care made it easier for SU to stay in contact with their social and family networks and to return to work even during treatment. Yet, the lack of distance to one's own personal background and social sphere, the lack of a given structure and of distractions, the feeling of isolation and the need for self-organisation also during acute crises were described as challenges by some SU, who were treated at home:

*“It is not always easy for someone like me, who doesn't have enough daily structure. Of course, the flexible [home treatment] team brings some routine into your life, depending on how often you need it” (SU, Department G)*.

Caregivers were quite ambivalent about the integration of everyday life in FIT64b models. On the one hand, they experienced home treatment as an advantage, as it allowed them to be present during therapeutic sessions and to contribute to the recovery of their kin. On the other hand, this gave them an additional responsibility that sometimes was described as a burden. Both patients and caregivers first had to get used to the intrusion of the hospital staff in their personal spaces and to the associated experienced loss of possibilities for retreat. Yet, as they became more acquainted with the benefits of outreach care, their initial reservations gradually diminished.

## Discussion

The main objective of the present work was to examine the impact of implementing an integrative, GTB-based model of psychiatric care on SU, caregivers and employees.

Based on the stakeholders' experiences, a logical diagram was developed (**Figure 3**) to illustrate the implementation process from its inputs to its outcomes. Although the underpinning Throughput-Model (**Figure 1**) is rather linear in structure, it includes contextual factors (e.g., legal frameworks, remuneration systems) and systemic effects (such as feedback mechanisms) (27, 28). Accordingly, by using the Throughput-Model we aimed at overcoming traditional evaluative approaches that reduce intervention outcomes to only few parameters (35), thus examining the impact that GTB-based FIT64b models may have on the broader context of the stakeholders' lives. As shown above, the provision of care did not only affect SU and their caregivers but it also influenced the overall treatment culture and ethos: It resulted into a changed practice of dealing with acute crisis situations among staff, leading to a more confident and autonomy-promoting attitude.

**Figure 4:**
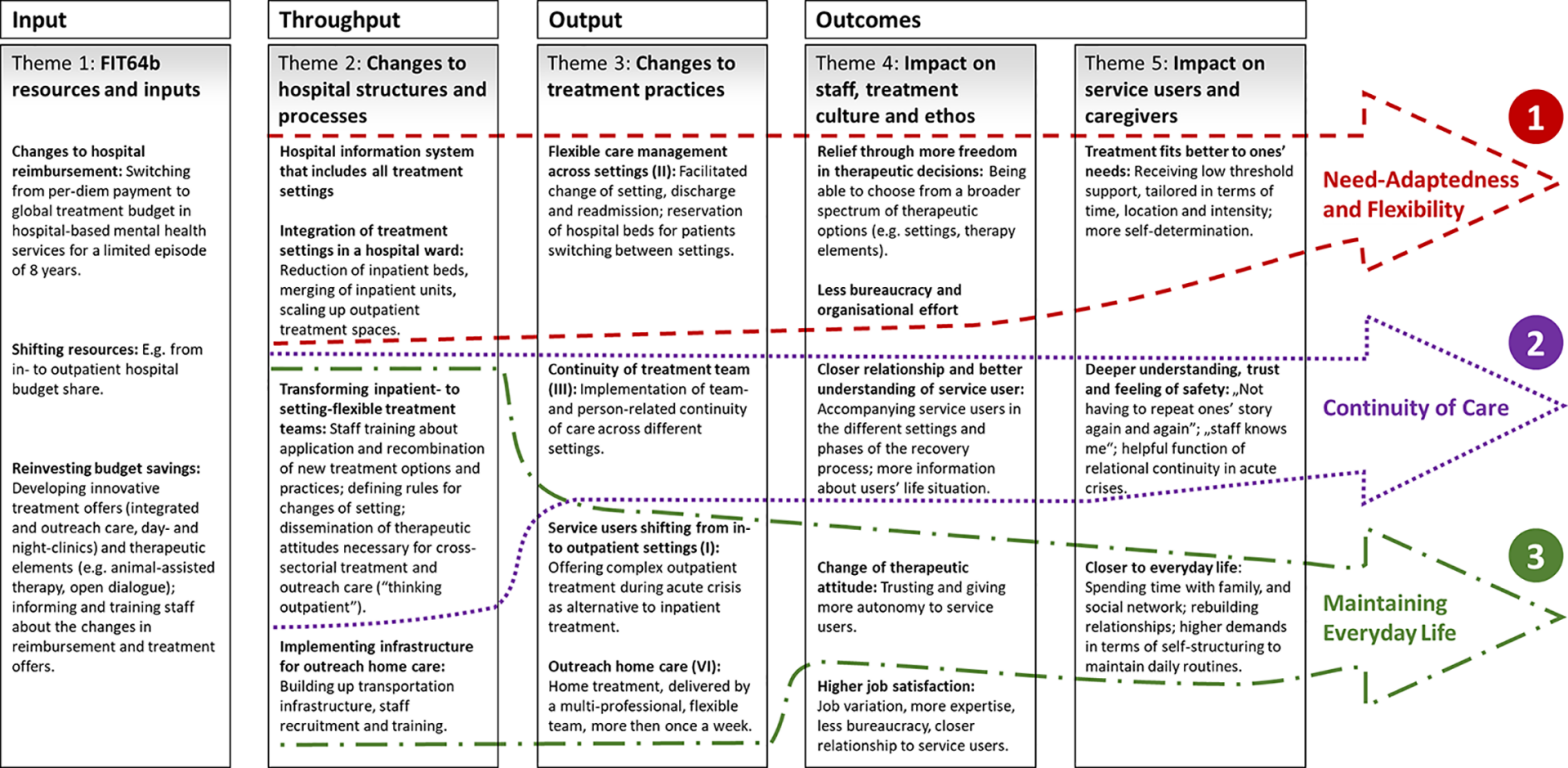
Logical diagram of FIT64b models integrating its major change mechanisms (numerals 1–3). I, II, III, VI: the Roman numerals refer to the key components of FIT64b. All FIT64b specific components can be found in **Table 1**.

In what follows, we first discuss the impact that a GTB may have on implementation and practice within FIT64b models. Second, we present key impact mechanisms within the developed logical diagram (**Figure 4**) by integrating the results of our qualitative analysis with the related literature. Third, we illuminate how the identified change mechanisms vary between the two prototypes of FIT64b implementation by taking into account both the semi-quantitative and the structural data of the involved hospitals.

### Impact of Global Budget Approaches

Our findings describe how integrated psychiatric care was gradually built-up, based on the financial securities provided by a GTB. This would not have been possible under the conditions of the common day- and performance-oriented reimbursement system of German hospital care (8). The GTB allowed to reallocate to outpatient settings the now unutilized inpatient hospital structures and to employ former inpatient staff in outpatient and outreach services (35). We showed that the re-allocation of FIT64b resources in proactive and preventive ways allowed to avoid intensive forms of treatment and thus, to save expenses on the long-term. These results align with findings by the British Medical Association stating that financing approaches similar to GTB, like capitation payment, encourage greater investment in the secondary or tertiary preventive and community-based care because they allow to flexibly allocate resources so as to produce the best possible outcomes for SU (6, 7).

### Modeling Key Change Mechanisms

With the development of the logical diagram (**Figure 3**) it turned out that almost each throughput factor can be connected *via* the outputs to the outcomes. This explains which aspects of the throughput are fundamentally responsible for which outcomes. **Figure 4** shows the previously developed logical diagram including three key change mechanisms. These are: (1) Need-Adaptedness and Flexibility, (2) Continuity of Care, and (3) Maintaining Everyday Life. In what follows, we discuss these three central lines of impact, including the related existing literature. This procedure adheres to the recommendations of the MRC framework for evaluating complex interventions, which indicates the necessity of both empirically and theoretically grounded modeling (20).

#### Need-Adaptedness and Flexibility

One key mechanism of our logical diagram is the positive impact of the increased flexibility of treatment on the need-adapted nature of care within FIT64b models (**Figure 4**). The integration of settings and teams and the simplification of bureaucratic processes allowed SU to swiftly shift between settings and, thus, to be treated more according to their needs. These findings align with the definition of flexibility put forward by other team-based care models such as the Dutch flexible ACT (36), which mainly relies on the idea of adapting the treatment intensity to the concrete needs of SU.

Our results thus show how the primarily economic flexibility of a GTB was directly passed onto the everyday structure of the services and to the SU themselves (6, 7): For instance, by eliminating the economic constraint of occupying inpatient beds, the hospital departments were able to keep spare beds available in case a SU needed to be readmitted. Our participants evaluated this as a significant increase in need-adaptedness.

#### Continuity of Care

Relational continuity emerged from our results as a fundamental factor of flexible and integrated psychiatric care according to §64b Social Code V (**Figure 4**). The transformation of setting-specific teams into setting-flexible ones supported the establishment of longer-term, trusting relationships between SU and teams (or team members). This, in turn, fostered feelings of trust and safety in SU (36). Our findings align with the ones of previous studies, which have shown a positive correlation between relational continuity and SU's clinical outcomes or satisfaction (37–39). In contrast, Giacco et al. (40) conclude from a one-year observational study that patients treated by the same psychiatrist in different settings do not show better outcomes than those treated by different clinicians. Based on our findings, we argue that SU benefit from relational continuity even beyond measurable clinical outcomes. On the one hand, recovery processes are complex and highly individual and therefore cannot be solely captured by clinical measures. On the other hand, both our and the already existing research emphasizes that many positive effects of a continuous therapeutic connection might be only measurable after a longer period of time (39, 41).

#### Maintaining Everyday Life

The third causal mechanism of our logical diagram describes how outpatient or outreach treatment services in moments of acute crisis may strengthen the SU' integration in their everyday life (**Figure 4**). This aligns with the results of several evaluation studies on CRT teams, confirming that SU prefer outreach programs over inpatient treatment (42). Another important finding is that all involved stakeholders initially have to get used to this form of treatment: Employees have to be sufficiently trained to be able to carry out home treatment safely and independently and the SU and their families have to get used to the staff “invading” their privacy (42). Yet, as much as SU and caregivers showed initial scepticism about new forms of outreach care, this scepticism mostly faded away during the course of treatment (13).

### Two Prototypes of Implementation

The comparison of semi-quantitative data between the psychiatric departments participating in the study demonstrates the heterogeneous implementation of FIT64b models across Germany. This heterogeneity especially emerges from the differences across the involved departments in implementing the FIT64b's key components (see **Table 3**) (12, 14). This is not surprising since the given legal framework includes very vague specifications regarding the concrete implementation. With the goal of systematizing these differences, we have derived from the results two prototypes of FIT64b implementation (see **Table 5**). Hospitals E, F, and J could not be included because they do not fully meet the characteristics of either prototype.

**Table 5:** Two prototypes of FIT64b implementation.

Characteristics:	Prototype:	I	II
Study departments/municipalities		A, B, C, D	G, H, I
Population Density^7^		low	high
Contract closing date; Start of FIT64b implementation		2013	2016
Budget share (%)^8^		100	<33
Existing experiences with a GTB^5,9^		+	
Reduction of hospital beds (occupancy) since introduction of a GTB ^5^		+	
Treatment D^2^, I^1^, and/or O^3^ in the same unit (ward, level etc.) ^6^		+++	+
Staff working in more than one SoT^4^ (%)		>66	>33
Outreach home care by I^1^- and D^2^-teams ^5^		+	
Corresponding outreach care model		ACT	CRT

GTB: Global Treatment Budget; ACT: Assertive Community Treatment; CRT: Crisis Resolution Teams; “ “: Not applicable; ^1^I, inpatient; ^2^D, day-patient; ^3^O, outpatient; ^4^SoT, Setting of treatment (outpatient, day-patient, inpatient); ^5^ Maximum expression of parameter = +; ^6^Maximum expression of parameter = ++++; ^7^a high population density is reached from a limit of 600 inhabitants per km²; ^8^ratio of health insurances (i.e., SU) who joined the contract according to §64b Social Code V in relation to the whole hospital budget (all SU treated in the hospital); ^9^existing experiences with a GTB according to §24 “Bundespflegesatzverordnung”, the §64b preceding legislation, valid from 2002-2009, offering hospitals a GTB for the duration of 5 years.

Hospitals aligning with type I are mostly located in rural areas, provide treatment according to FIT64b to all SU (100% budget share) and have collected several years of experiences with similar models of care and reimbursement. Since hospital routines were entirely switched to FIT64b, changes in health care provision are more comprehensive in these hospital departments (especially departments A and B): Out-, day-, and inpatient settings are integrated both in terms of spaces and personnel in almost all units of these departments. Thus, relational continuity is highly implemented and also partially extends into outreach care. Outreach care is predominantly provided over longer periods of time, with rather low treatment intensity, and thus most likely aligns with ACT teams. In general, extended catchment areas with large average distances between hospitals and SU's homes make the implementation of an intensive outreach treatment model hardly feasible (1).

Study departments of type II are situated in urban areas. They did not have previous experiences with a GTB or similar models of care. Those departments only treat a small percentage of SU according to FIT64b (budget share of less than 33%), whereas the vast majority of SU receives treatment as usual (budget share of at least 67%). Thus, two different models of care are kept running simultaneously, leading to friction losses and to limited degrees of implementation of the FIT64b specific components (15). Outreach care is here usually set up in the form of separate teams, providing a rather short-term, acute care, which is comparable to the CRT model (43). Consequently, there is only a slight continuity of treatment teams from the outreach to the inpatient setting.

One of the main reasons for the limited participation of health insurances and thus for the underdevelopment of FIT64b models in urban catchment areas is the problem of risk adjustment of capitated or global budgets (44). In metropolitan areas there is a much higher exchange of SU between neighboring catchment areas. If a SU “belonging” to the catchment area of a capitated hospital X is treated in another hospital Y, this complicates reimbursement, thus making the implementation of FIT64b models more challenging than in rural areas.

To summarize the differences between the two prototypes with regard to the previously identified impact mechanisms, it can be concluded that 1) hospitals which contracted their entire budget as a FIT64b model do provide a strong manifestation of all three impact mechanisms (see **Figure 4**), whereas 2) hospitals which negotiated a FIT64b model only for a small budget share (less than 33%) have a focus on keeping SU out of the hospital, i.e., maintaining their everyday life.

### Strengths and Limitations

A strength of this process evaluation study is that the impacting mechanisms of FIT64b were modeled both empirically and theoretically. This arguably, leads to a realistic understanding of FIT64b models and their implementation (20). A blind spot of this study may lie in the fact that the outcome evaluation was performed prior to the process evaluation (20). This goes against the MRC Guidelines on the evaluation of complex interventions, which recommend to first explore change mechanisms (process), in order to support the selection of measures suitable for outcome evaluation (20). We acknowledge this limitation and yet we believe that, since we used primarily qualitative and iterative analysis methods, we could still achieve an integrated form of evaluation for process and outcome.

A further limitation might be a possible selection bias, since the statements made by the study participants revealed a rather consistently positive view about GTB and FIT64b. One might indeed argue that possible adverse effects of GTBs such as “cherry picking” low-risk SU, “dumping” high-risk ones or an under-provision in order to minimize costs haven't been properly represented in the outcomes (7). Indeed, such adverse effects might be captured mainly by outsider perspectives, e.g., by stakeholders and hospitals without FIT64b models, which were not included in the study. However, since all stakeholders have also named several barriers to the implementation as well as the problematic effects of FIT64b models, we believe that we can confidently exclude the presence of such bias and that we have presented a rather balanced pictured of stakeholders' experiences.

The overall presentation of the SU and carers' experiences is very condensed within the described categories. For instance, we did not differentiate between short-, intermediate- and long-term outcomes of FIT64b models. Besides, SU were not considered as Input- and Throughput-factors in the “applied” Throughput-Model. This would have been of crucial importance, as the legal framework of FIT64b explicitly demands to strengthen patient orientation. In fact, our central concern in this study was to investigate the implementation process and basic change mechanisms of the care model mostly from a staff perspective. In the ongoing follow-up study “PsychCare” (2017 - 2020) these critical points are addressed, by using a co-productive methodology (45). For this purpose, so-called EEG (“experiential expert generated”)-PREMS are currently being developed. These in turn aim at improving the ecological validity of the logical diagram and its inherent change mechanisms from the SU' perspective.

## Conclusions

The change from a daily- and performance-based to a lump-sum hospital payment across all settings (GTB) can be regarded as a key driver for the further development of psychiatric inpatient services toward a more flexible, integrative, ambulatory, and region-adapted treatment.

Besides, remuneration *via* an annual lump-sum eliminates the economic constraint that leads hospitals to fully occupy resource-intensive inpatient treatment places. In return, the incentive to act in a preventive and long-term resource-saving manner allows for low-threshold, outpatient and outreach services to be set up.

These changes in hospital financing and service provision lead to complex impacts on the stakeholders, which may not solely be captured by existing clinical outcomes. Key impacts of this care model are the improvement of need-adaptedness, relational continuity, and everyday-life orientation of treatment.

## Data Availability Statement

The datasets underlying the current study are not publicly available due to the used data protection declaration and the nature of qualitative interviews where individual participants could be possibly identified. Parts of the dataset are available from the research group on reasonable request.

## Ethics Statement

The studies involving human participants were reviewed and approved by Ethics Committee of Medical Chamber Brandenburg, Cottbus, Germany [2016, No. S 7 (a)]. The patients/participants provided their written informed consent to participate in this study.

## Author Contributions

JS wrote the first draft of the manuscript. JS, LG, SP, and MH modified successive drafts. JS and SP were mainly responsible for development of the logical diagram. SP and MH contributed to the study design. All authors contributed to and have approved the final manuscript.

## Funding

The authors received a financial grant from nine hospital government bodies interested in the evaluation of their own clinical projects for the research, authorship, and publication of this article. The funding bodies were represented in the study's steering committee by their heads of psychiatric departments. The steering committee was in control of the study's budget. It supervised the development of the study design, but there was no influence on the collection, interpretation or representation of data.

## Conflict of Interest

The authors declare that the research was conducted in the absence of any commercial or financial relationships that could be construed as a potential conflict of interest.

## Abbreviations

ACT, Assertive Community Treatment; CoC, Continuity of Care; CRT, Crisis Resolution Team; EvaMod64b, Evaluation of Models according to §64b of Social Code V; FIT64b, Flexible and Integrated Treatment according to §64b of Social Code V; HIS, Hospital Information System; MRC, UK Medical Research Council; PREMs, Patient Reported Experience Measures; SU, Service User.
