# Supplementary material for: Changes in German Mental Health Care by Implementing a Global Treatment Budget—A Mixed-Method Process Evaluation Study
Source: Front Psychiatry. 2020 May 25;11:426. doi: 10.3389/fpsyt.2020.00426 (PMC7261866; doi:10.3389/fpsyt.2020.00426)
Supplement: Supplementary file 1 [file Table_1.docx]

***Supplementary Material***

**Table S1**. Exemplary sampling plan (here: focus group with the topic “Changes in daily routines of FIT64b models“)

| **Stakeholder group** | Description of [recruitment strategy](https://www.linguee.de/englisch-deutsch/uebersetzung/recruitment+strategy.html) |
| --- | --- |
| Service user | Three service users should be recruited in total. The balance especially regarding gender, level of education and age (elderly and very young patients are welcome, too) should be considered. Illness severity and duration of illness should diverge (from serious/long to less severe/short illness experiences). Is it possible to select a service user with migration background? Each service user should have made extensive experiences with the treatment according to FIT64b (including the attached FIT64b specific components); they should have had a low or high benefit from the treatment according to FIT64b (according to the assessment of the study employees on site). The selection of service users who also know other treatment situations (from other hospitals or in the same hospital before the introduction of the FIT64b model), could be helpful. Service users who proactively/independently take responsibility for their own recovery process and service users, who had shown themselves rather resistant and defensive in relation to previous psychiatric treatment (for instance users who have been hospitalized against their will and who were victims of coercion and violence during a psychiatric treatment episode) are equally to be involved. Service users who had experienced a flexible, cross-sectorial and/or home-treatment are of particular interest. Participants should be able to be questioned within a group setting and have sufficient German language skills. |
| Caregiver | Two caregivers (of all genders) of service users, who received treatment according to FIT64b models, should be recruited. They should *not* be family members of service users who are involved in the focus-group (trialogical principle). Siblings, parents and partners may be selected. Caregivers who live together with their ill family member should be preferred. Caregivers who have experienced other treatment situations (from other hospitals or in the same hospital *before* the introduction of the FIT64b model), could be helpful. Is it possible to recruit a caregiver with a migration background? |
| Staff | There should be at least 2 employees within the focus group, of different genders and different (long/short or rather intensive) experience with the treatment according to FIT64b models. The extent of the professional experience should be considered (long time employees have sometimes another view on structures and processes than “newcomers”). Employees who have experienced other treatment situations (from other hospitals or in the same hospital *before* the introduction of the FIT64b model), could be helpful. The employees should belong to different professional groups. They should have collected practical experiences with the treatment according to FIT64b. This means that they should rather be “employees on site” (versus staff in managing positions). Employees, who know the caregivers and/or service users who were selected to participate in the focus group (responsible treatment team) are preferred. |

**Table S2.** Exemplary interview guide (here: focus group with the topic “Changes in daily routines of FIT64b models“)

| **Chances in everyday life and routines**  In your hospital, a so-called FIT64b model was implemented during this year. This is a new form of treatment, which should allow the employees to support service users and their caregivers in a more flexible and need-oriented way. We want to examine the effects of this FIT64b model on everyday life and treatment situations. To get the most complete picture possible, we are interested in the viewpoint of all stakeholders of a treatment situation, which means service users, caregivers and employees.  Questions to make clear in advance:   - Did any of you receive treatment or has been employed in this hospital *before* 2016? - Did any of of you receive treatment or has been employed in another hospital? | | | | |
| --- | --- | --- | --- | --- |
|  | Key question/ Narrative impulse | Aspects to check | Concretising questions | Questions to maintain or control the conversation |
| 1. | How did the provided treatment change in this hospital during this year? How do you experience the treatment here in this house? | General assessment and check of the patient-relevant FIT64b specific components (I-VII and X), as well as concretise component I-XI | - Characterize the treatment received by the team with using only three catchwords/terms  - Do you have the impression, that the treatment in this hospital is tailored to your needs (living situation)/ to the needs (living situation) of the service user? In comparison with other offers/ institutions?  - (If yes) what is done in this hospital to react adequately to the needs (e.g. living situation) of the service user? | **In which moment regarding your course of treatment/ in which way did it become clear? In which situation did it become clear?**  **How do you experience that?**  Could you tell more about it?  How did you experience it?  How was it for yourself?  How do you see that?  Could you please provide details about this?  Could you give an example for that?  Could you concretise that?  How did this happen?  Please tell me how it was? |
| 2. | **(Using a vignette/hand-out)**  Which parts of FIT64b models are already implemented in this house? | General stimulus and check of the experiences made within the hospital | -Name benefits and disadvantages for both treatment pathways for an imagined service user.  -In which situation is which treatment model helpful or rather disadvantageous?  -What is facilitated by both models, what makes them more complicated? |  |
| 3. | Within FIT64 models, it is possible to flexibly switch between different settings of treatment. How did this change in this hospital during 2016? | Flexible care management across settings (II), therapeutic group sessions across all settings (V), cooperation across sectors (X), continuity of treatment team (III) | -Could you describe a situation in which it was important that the setting of treatment could be changed with flexibility? Which benefits and disadvantages are associated with flexible changing of treatment setting?  -Could you describe a situation in which it was important that the same treatment team has supported you across different settings? Was there a situation in which this form of continuity had disadvantageous effects?  -Can you visit the same therapeutic groups while being treated within different hospital settings in this hospital? If yes, describe why it is (not) important to take part in cross-sectorial therapeutic group sessions. What are the disadvantages?  -How do you experience the cooperation of the hospital employees with other institutions beyond the hospital (e.g. assisted living, independent outpatient therapists, employment office)? |  |
| 4. | There is a slogan in the psychiatric treatment: “Outpatient before inpatient”: Do you have the impression that the treatment in this hospital has been shifting gradually from in- to outpatient setting? | Shifting in- to outpatient setting (I), Home treatment (VI), accessibility of services (VIII) | -Which benefits does an outpatient treatment have for you (did you benefit from it)?  -What are the disadvantages or challenges?  - How do you experience home treatment (if available)? Which benefits or disadvantages has the treatment at home?  - Is there also a treatment at home in phases of acute illness (benefits and disadvantages)? |  |
| 5. | Within FIT64b models, relatives and other caregivers should be more strongly involved in the treatment. Regarding this, did something change in this year? | Involvement of cares (VII) | -In which way does this hospital include relatives and other caregivers in the treatment process?  - How helpful is it?  - When is it not so helpful?  - Which preconditions does the hospital need to more strongly involve relatives and other caregivers in the treatment process? |  |
